# Identifying the Association Between Older Adults' Characteristics and Their Health-Related Outcomes in a Transition Care Setting: A Retrospective Audit

**DOI:** 10.3389/fpubh.2021.688640

**Published:** 2021-06-28

**Authors:** Jo-Aine Hang, Jacqueline Francis-Coad, Chiara Naseri, Angela Jacques, Nicholas Waldron, Kate Purslowe, Anne-Marie Hill

**Affiliations:** ^1^Curtin School of Allied Health, Curtin University, Perth, WA, Australia; ^2^Department of Aged Care and Rehabilitation, Armadale Kelmscott Memorial Hospital, East Metropolitan Health Service, Armadale, WA, Australia; ^3^Amana Living Inc., Perth, WA, Australia

**Keywords:** aged, continuity of patient care, independent living, intermediate care, outcome assessment (health care)

## Abstract

**Introduction:** Continued evaluation of Transition Care Programs (TCP) is essential to improving older adults' outcomes and can guide which older adults may benefit from undertaking TCP. The aim of this study was to audit a transition care service to identify the association between the characteristics of older adults undertaking a facility-based TCP and (i) discharge destination and (ii) functional improvement.

**Materials and methods:** An audit (*n* = 169) of older adults aged 60 years and above who completed a facility-based TCP in Australia was conducted. Outcomes audited were performance of activities of daily living (ADL) measured using the Modified Barthel Index (MBI) and discharge destination. Data were analyzed using logistic regression and linear mixed modeling.

**Results:** Older adults [mean age 84.2 (±8.3) years] had a median TCP stay of 38 days. Fifty-four older adults (32.0%) were discharged home, 20 (11.8%) were readmitted to hospital and 93 (55%) were admitted to permanent residential aged care. Having no cognitive impairment [OR = 0.41 (95% CI 0.18-0.93)], being independent with ADL at admission [OR = 0.41 (95% CI 0.16-1.00)] and a pre-planned team goal of home discharge [OR = 24.98 (95% CI 5.47-114.15)] was significantly associated with discharge home. Cases discharged home showed greater improvement in functional ability [MBI 21.3 points (95% CI 17.0-25.6)] compared to cases discharged to other destinations [MBI 9.6 points (95% CI 6.5-12.7)].

**Conclusion:** Auditing a facility-based TCP identified that older adults who were independent in ADL and had good cognitive levels were more likely to be discharged home. Older adults with cognitive impairment also made clinically significant functional improvements.

## Introduction

Older adults requiring an episode of hospitalization are more susceptible to developing new functional deficits during their stay, in part due to prior decline in functional ability (1, 2). Thirty-five to 60% of older adults demonstrate functional decline at hospital discharge, making it challenging for them to successfully return home (1, 2). In order to bridge the gap between hospital and home and prevent premature admission into residential aged care (RAC), Australia established a step-down rehabilitation service known as “Transition Care” (3). Other countries also provide transitional programs for at risk patients, usually older adults, after hospital discharge known as “Intermediate Care” in United Kingdom (4) and “Skilled Nursing” in United States (5). The goal of providing Transition Care Programs (TCP) in either facility-based or community settings is to deliver ongoing multidisciplinary rehabilitation for older adults immediately after an acute hospital admission (3). TCP aim to rehabilitate older adults to regain their functional independence while enabling them and family members to plan for discharge home or transfer to RAC if required (3).

However, there is mixed evidence about whether older adults who complete TCP demonstrate improved physical or cognitive function (3, 6). A recent large national study in Australia found that only 38% of older adults who completed a TCP demonstrated improvement in functional independence (3). Proportions and predictors of discharge home from TCPs varies within and between countries, with ~50% of older adults in Australia being discharged home compared 80% in Norway (3, 6). Characteristics associated with discharge home have been found to include absence of cognitive impairment, being younger, and having better performance of ADL (3, 6). This variation is thought to be due in part to the differences in the setting, type and intensity of TCP provided and type of population admitted to each TCP, including older adults' functional ability at admission (3, 4, 6, 7). A key difference in program settings is that some TCP are delivered in the home using a re-ablement model while others are delivered in facilities (3, 4). Older adults requiring a higher level of care and those with less social support are often admitted to a facility-based TCP as there is 24 h care support available. A national audit in Australia found that Western Australia provided TCP mainly in facility-based settings compared to other states (3). There is also wide variation between countries in health outcomes achieved from TCP (3, 4), in particular the success of the program in reducing admission to RAC. Therefore, continued evaluation of TCP is essential to assist understanding what type of TCP improve older adults' outcomes, including functional ability and eventual discharge destination and allocating scarce health resources (4). Program evaluation is also important to measure effectiveness and quality.

Audit and feedback (A&F) enables clinical care staff and organizations to evaluate their program performance against evidence-based guidelines and to compare programs within and across services (8). In Western Australia one such organization delivers TCP in both facility and home settings. This organization decided to conduct an audit to use as a comparison with current TCP and with similar programs across the broader health setting. This aimed to form a foundation from which to improve TCP and inform future allocation of scarce health resources. The aim of the study was to conduct a retrospective audit to identify the association between older adult characteristics and (i) discharge destination, and (ii) functional improvement in a Transition Care (TC) facility-based setting.

## Materials and Methods

### Design

We conducted a secondary data analysis of de-identified data from a cohort of older adults who had undertaken facility-based TC rehabilitation. Cases were identified from electronic medical records, and additional data collected by hand searching archived paper medical files.

### Ethical Considerations

This study was approved by Curtin University Human Research Ethics Committee and the participating organization's clinical governance committee (Amana Living Inc.). A de-identified data base was supplied by the organization.

### Setting and Sample

All cases admitted to a 47 bed TC facility in metropolitan Perth Western Australia from January 1st to October 31st 2018 were reviewed. Cases consisted of older adults aged 60 years and above, who were admitted to the facility to undertake a TCP during the study period. Cases were excluded if the older adult was admitted for palliative care, or for <2 weeks duration.

The facility health professional staffing included a manager, registered nurse, physiotherapist, social worker, and an occupational therapist. A general practitioner, speech pathologist, nutritionist, and podiatrist visited as required. Care assistant staff also assisted clients with daily personal care. The TCP included physiotherapy for functional and mobility training, occupational therapy for cognitive activities and home visits, and social work for discharge planning and care support at home.

### Variables

Data recorded for each case at time of TC admission were collected. These variables were age, gender, socioeconomic status measured using Index of Relative Socio-Economic Advantage and Disadvantage (IRSAD) 2016 (9), length of stay (LOS) in hospital prior to admission to the TC facility, whether assistance for ADL or IADL was required prior to hospital admission, living situation prior to hospital admission (alone or with others), use of walking aid, primary diagnosis, number of medications on admission, falls history (prior to TC facility admission), presence of cognitive impairment, presence of depression, and malnourishment (see Supplementary Table 1). The primary diagnosis for cases was categorized as neurological, cardiorespiratory, orthopedic, general medicine/surgical, or geriatric conditions. Where case notes recorded a diagnosis of a mental health condition by the psychiatric team in addition to the primary diagnosis, this was categorized as a separate independent variable. Receiving assistance prior to admission was categorized as receiving assistance with activities of daily living (ADL) using the Katz Index (10) or instrumental activities of daily living (IADL) using the Lawton scale (11).

Each case also had recorded a “pre-planned discharge destination,” which was defined as the participant's expected discharge destination on completion of TCP and was pre-determined by the multidisciplinary team in the hospital prior to Transition Care admission. This pre-planned discharge destination plan was provided to the TCP team at point of admission. The plans nominated the older adult to be discharged after completing their TCP to (i) home or (ii) RAC facility. Previous research suggests that older adults with limited social support, multiple hospital readmissions over the 12 months, high levels of care needs, and multiple medical comorbidities were the most likely candidates to have a pre-planned discharge to residential aged care (7).

### Outcomes

The primary outcomes were:

(i) discharge destination categorized as home, RAC facility, or hospital readmission. For group comparisons, discharge was defined as home vs. not home.
(ii) functional ability measured using:

- the Modified Barthel Index (MBI) which measures the older adult's ability to complete personal care tasks including showering and toileting (score 0 to 100—higher scores indicating better ADL performance) (12, 13). This was measured at admission and discharge from TCP to determine changes in functional ability that occurred during TCP;
- whether or not the older adult received carer assistance either for personal care tasks, such as showering or toileting (Katz ADL scale) (10, 14) or for instrumental care tasks such as shopping or cleaning (Lawton's scale) (11) prior to hospitalization and when discharged home after TCP.

### Statistical Analysis

Data were summarized using descriptive statistics (frequency distributions for categorical data and means and standard deviations or medians and interquartile ranges (IQR) for continuous data). Group comparisons between cases who were discharged home and those who were discharged elsewhere (RAC, hospital) were made using Chi squared or Fisher Exact tests, as appropriate, for categorical data and *t*-tests or Mann-Whitney *U*-tests for continuous data, depending on normality of the distribution. Logistic regression was used to determine case characteristics that were predictors of being discharged home. Univariable logistic regression was undertaken and univariately significant variables (with a *p*-value of < 0.05) were entered into a multivariable model. Linear mixed modeling was used to determine predictors of improvement in functional ability during their stay in TCP (measured by MBI) from admission to discharge. Analyses were completed using IBM SPSS version 24.0 (Armonk, NY) and STATA version 16.1 (StataCorp. 2019. Stata Statistical Software: Release 16. College Station, TX: StataCorp LLC).

The sample size was determined based on the minimal clinically important difference (MCID) that has been established for the MBI (15). It was determined that a sample of *n* = 166 cases has 80% power (alpha = 0.05) to detect a standardized mean difference (effect size dz) of 0.22 (based on mean ± SD = 1.85 ± 8.5) in a matched pairs *t*-test to compare pre-post measures in a single group (using statistical power analysis program G^*^Power 3.1.9.2) (16). The sample was drawn from all cases admitted to a 47 bed TC facility in metropolitan Perth Western Australia from January 1st to October 31st 2018.

## Results

### Case Characteristics

There were *n* = 169 cases extracted from the database. All cases had a discharge destination and an admission MBI recorded and *n* = 133 cases had a discharge MBI score. Baseline characteristics of cases are presented in Supplementary Table 1. Approximately one third (*n* = 56) of the cases had a primary orthopedic diagnosis (this included cases having total hip replacement surgery after hip fracture).

### Discharge Destination

**Figure 1:**
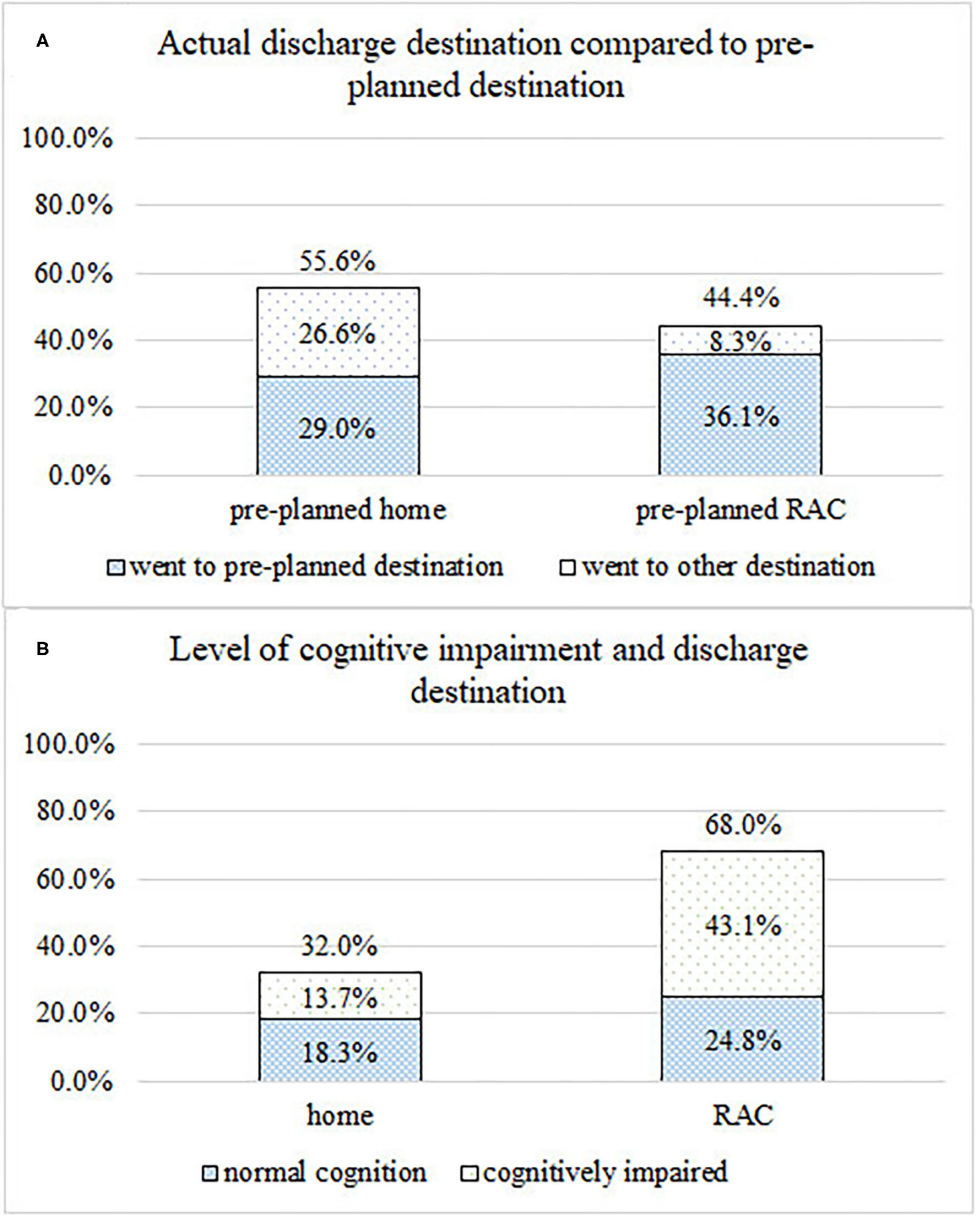
**(A)** Proportion of cases actual discharge destination compared to pre-planned discharge destination. **(B)** Number of cases discharged home compared to other discharge destinations, categorized by presence or absence of cognitive impairment.

There were 32.0% (*n* = 54) cases discharged home, 55.0% (*n* = 93) admitted to RAC, and 11.8% (*n* = 20) readmitted to hospital. Cases who were discharged to their pre-planned discharge destination compared to their actual discharge destination are presented in Figure 1A. Of those who had a pre-planned destination of home, 52.1% (*n* = 49) went home and 34.0% (*n* = 32) went to residential care while 81.3% (*n* = 61) of those whose plan was for discharge to RAC were admitted to RAC. Percentages of cases categorized by level of cognitive impairment who were discharged home compared to other settings are presented in Figure 1B. Of those who discharged home (*n* = 54), there was a statistically significant increase in the number of older adults requiring assistance with ADL at discharge (*n* = 32) compared to admission (*n* = 14) (*p* = 0.027). There was no statistical difference for the number of older adults requiring assistance with IADL at discharge (*n* = 47) compared to admission (*n* = 53) (*p* = 0.130).

Characteristics of cases discharged home compared to other settings are presented in Table 1. Univariable analyses for the outcome of discharge home are presented in supporting information online Supplementary Table 2. Cases who were receiving assistance with ADL prior to hospitalization, had cognitive impairment or had a diagnosis of a cardiorespiratory condition were significantly less likely to be discharged home. Cases who had a history of falls prior to hospital admission or had a pre-planned discharge destination home were more likely to be discharged home. Adjusted multivariable modeling for the outcome of discharge home is presented in Table 2. Older adults were significantly more likely to be discharged home if they did not require ADL support at home prior to hospitalization, had an orthopedic diagnosis, had no cognitive impairment and had an initial team plan that aimed for discharge home.

**Table 1:** Older adults' characteristics—group comparisons for cases discharged home compared to other discharge destination.

**Cases characteristics**	**Discharge destination**		***P*-value**
	**Home *n (%)***	**Other *n (%)***	
	***n = 54*^**†**^**	***n = 115*^**†**^**	
Age (mean, SD)	82.5 (7.8)	85.1 (8.4)	0.030^*^
**Gender**			
Female	31 (57.4)	72 (62.6)	0.518
Male	23 (42.6)	43 (37.4)	
**Socioeconomic status, IRSAD**^**‡**^			
I	9 (16.7)	6 (5.22)	0.045^*^
II	13 (24.1)	36 (31.3)	
III	32 (59.2)	73 (63.5)	
**Assistance with ADL**			
Yes	14 (25.9)	49 (42.6)	0.036^*^
No	40 (74.1)	66 (57.4)	
**Assistance with IADL**			
Yes	47 (87.0)	100 (87.0)	0.988
No	7 (13.0)	15 (13.0)	
**Uses walking frame**			
Yes	37 (68.5)	85 (73.9)	0.466
No	17 (31.5)	30 (26.1)	
**Living situation**			
Alone	33 (61.1)	62 (53.9)	0.379
With others	21 (38.9)	53 (46.1)	
Hospital LOS (median, IQR)	31.5 (21.0-42.0)	34.0 (22.0-50.0)	0.419
**Took** **≥** **7 medications**			
Yes	45 (86.5)	91 (79.8)	0.297
No	7 (13.5)	23 (20.2)	
**Primary diagnoses**			
Neurological	6 (11.1)	19 (16.5)	0.002^*^
Cardiorespiratory	3 (5.5)	27 (23.5)	
Orthopedic	28 (51.9)	28 (24.3)	
General medicine/surgical	10 (18.5)	18 (15.7)	
Geriatric related^§^	7 (13.0)	23 (20.0)	
**Mental health diagnosis**^**¶**^			
Yes	9 (16.7)	25 (21.7)	0.443
No	45 (83.3)	90 (78.3)	
**History of falls**			
Yes	50 (94.3)	87 (77.7)	0.008^*^
No	3 (5.7)	25 (22.3)	
**Presence of cognitive impairment**^**††**^			
Yes	21 (42.9)	66 (63.5)	0.016^*^
No	28 (57.1)	38 (36.5)	
**Incontinence**			
Yes	29 (55.8)	73 (65.8)	0.219
No	23 (44.2)	38 (34.2)	
**Presence of depressive symptoms**^**‡‡**^			
Yes	19 (61.3)	42 (63.6)	0.824
No	12 (38.7)	24 (36.4)	
**Malnourished**^**§§**^			
Yes	30 (65.2)	68 (73.1)	0.336
No	16 (34.8)	25 (26.9)	
**Discharged to pre-planned discharge destination**			
Yes	50 (92.6)	61 (53.0)	<0.001^*^
No	4 (7.4)	54 (47.0)	

*ADL, activities of daily living; IADL, instrumental activities of daily living; LOS, length of stay*.

* *p < 0.05*.

† *All data are reported as n (%) unless otherwise stated. Where data not = 100%, data are missing*.

‡ *IRSAD, The Index of Relative Socio-Economic Advantage and Disadvantage 2016, where I = most disadvantaged socioeconomic area and III = most advantaged socio-economic area*.

§ *Includes poor balance, malnutrition, frailty, polypharmacy, incontinence, delirium, and fall risk (17)*.

¶ *Co-morbidities, includes depression, anxiety, suicidal ideation, post-traumatic stress disorder, low mood, adjustment disorder, and paranoia*.

†† *Measured using Mini Mental State Examination (MMSE), scored ≤ 23/30 at admission (18, 19)*.

‡‡ *Measured using Geriatric Depression Scale, scored ≥ 6 points indicating presence of depression (20)*.

§§ *Measured using Mini Nutritional Assessment Short-Form (MNA-SF), scored ≤ 11 points indicating malnourishment (21)*.

**Table 2:** Multivariable analysis for Outcome = home (FINAL).

**Predictor^**†‡**^**	**Univariate**			**Multivariable**		
**(Case characteristics)**	**OR**	**95% CI**	***P*-value**	**AOR**	**95% CI**	***P*-value**
Assistance with ADL	0.47	0.23-0.96	0.038	0.41	0.16-1.00	0.049
Primary—Orthopedic diagnosis	3.35	1.69-6.62	0.009	3.63	1.51-8.68	0.004
Presence of cognitive impairment	0.43	0.22-0.86	0.017	0.41	0.18-0.93	0.033
Discharged to pre-planned discharge destination	5.51	2.68-11.32	<0.001	24.98	5.47-114.15	<0.001

*ADL, activities of daily living; AOR, adjusted odds ratio; CI, confidence intervals; OR, odds ratio*.

† *Cases baseline data (n = 169) other than presence of cognitive impairment (n = 153)*.

‡ *See full model including list of covariates (independent variables) in Supplementary Table 2 Multivariable analysis for Outcome = Discharge home*.

### Functional Ability

There was a significant mean difference of 13.7 points (95% CI 11.0-16.4, *p* < 0.001) between cases' functional ability at admission [MBI 48.8 points (95% CI 45.4-52.2)] and discharge [MBI 62.5 points (95% CI 58.9-66.1)]. Cases who were discharged home had a mean improvement in functional ability (MBI) 21.3 points (95% CI 17.0-25.6, *p* < 0.001) as compared to cases who were discharged to other destination who made a mean improvement of (MBI) 9.6 points (95% CI 6.5-12.7, *p* < 0.001).

Comparisons of improvements in functional ability by age and presence or absence of cognitive impairment are presented in Table 3, (see also Supplementary Figure 1). There was no significant difference between the improvement made by the four groups [coefficient 7.4 (95% CI −5.603-20.496) (*p* = 0.263)]. However, there were significant within groups improvements made between admission and discharge.

**Table 3:** Age and cognition in predicting functional improvement.

**Age group (years)**	**Presence of cognitive impairment^**†**^**	**MBI^**‡**^ pre**	**95% CI**	**MBI post**	**95% CI**	**Mean difference**	**95% CI**	***P*-value**
60-79^§^	No	52	42.5-61.5	71.1	60.9-81.3	19.1	10.9-27.4	<0.001
	Yes	40.2	32.5-47.8	52.8	44.7-60.8	12.6	6.11-19.1	<0.001
≥80^§^	No	55.1	47.9-62.4	68.5	60.3-76.6	13.3	6.63-20.0	<0.001
	Yes	50.3	45.6-54.9	64.5	59.6-69.4	14.2	10.3-18.1	<0.001

*CI, confidence intervals; MBI, Modified Barthel Index*.

† *Measured using Mini Mental State Examination (MMSE), scored ≤ 23/30 at admission (18, 19)*.

‡ *Measured performance of activities of daily living (ADL), range 0-100, higher score indicates greater level of independence (12, 13)*.

§ *Analyzed using linear mixed modeling to estimate the effect of variability in age and cognition on functional improvement*.

## Discussion

In this Australian cohort of older adults who completed a facility-based TCP, 32% were discharged home. Being independent with ADL at home prior to hospitalization, having an orthopedic diagnosis, having no cognitive impairment and a pre-planned goal of discharge home were significantly associated with returning home. Higher levels of independence with ADL at admission to TC facilities have previously been found to predict discharge home in participants with various diagnoses including stroke (6, 22). A randomized controlled trial conducted in Norway found no difference between the proportions of older adults discharged home from an IC facility compared to those who stayed in hospital, but found that more older adults who were discharged from an IC facility lived independently without home health care services (23). In contrast, a national study in Australia found that older adults who were undertaking TCP in a facility-based setting were more likely to be discharged to RAC and less likely to improve their functional abilities compared to those who undertake TCP in community-based settings (3). Since the goal of TCP is to promote discharge home and avoid RAC admissions, these differences between countries suggest that further evaluation of TCP is required to understand what programs are most effective. Approximately 60% of the older adults who were discharged to RAC had cognitive impairment which accords with other studies that found cognitive impairment significantly reduced the likelihood of older adults returning home (3, 6, 24).

In this TC setting, a health professional team from the discharging hospitals provided a discharge plan, based on assessment of the older adult prior to their transfer to the TC facility discharge. However, only 64% of these discharge plans were accurate predictors of the actual discharge destination achieved by older adults following TCP. This finding concurs with another Australian retrospective study that reported 60% of these types of predictive discharge plans were accurate (25). Other studies have suggested that that having a pre-determined plan influences TCP staff and older adults' motivation and behaviors in engaging with rehabilitation (25, 26). This may negatively impact outcomes for older adults if their discharge plan was pre-determined as admission to RAC.

The cohort's levels of independence in ADL improved significantly and above a MCID through undertaking facility-based TCP. Improvement in ADL has been shown to vary across TCP settings with a range of between −9.26 points to 28 points (6, 27, 28). This could be due to differences in therapy duration, type of rehabilitation provided or the number of rehabilitation staff available, as suggested by a previous study that reported higher staff ratios were a significant predictor of functional improvement (29). There was no significant difference in the amount of improvement in functional ability based on age and cognitive impairment. However, adults in an older age range (80 years and over) with cognitive impairment had a lower functional level on admission. Some older adults discharged home were provided with extra personal and social support compared to admission, indicating that recovery to pre-admission levels might not have occurred. Additionally, older adults with cognitive impairment are at increased risk of experiencing adverse effects of hospitalization, such as functional decline, and are less likely to recover to their previous functional ability (30).

Findings from this audit suggest that TCP rehabilitation may need to be more tailored for older adults with cognitive impairment (31, 32) or more comprehensive home support services are required after discharge from TCP (33). Investigation into whether programs are effectively targeted to older adults with cognitive impairment is important as many older adults, including those with dementia, want to remain in their own home and currently only 5% lived in shared accommodation (34). Since motivation may affect engagement in rehabilitation, facility-based TCP should also consider a policy of engaging all older adults in their family to aim for discharge home as a first option rather than pre-plan for discharge to RAC.

### Strengths and Limitations of the Research

Our audit was strengthened by being conducted by the researchers in collaboration with the organization, as it is known that audits conducted by people who want change are more likely to result in change (35). A further audit of home-based TCP in the organization would be of value to compare patients' health outcomes and use of resources. The audit provided a detailed summary of a cohort of older adults that attended a facility-based TCP, evaluating what proportions were successfully discharged home, together with comparison of levels of functional improvement. Case notes were able to be examined in detail as well as electronic records. Although this audit was conducted at one facility it provides useful information on the characteristics of older adults undertaking facility-based TCP in Australia. While admission criteria for all Australian TCP are similar, heterogeneity between settings is likely to be present. There are limited studies that have specifically examined facility-based TCP. These findings may be useful as a comparison for other facility-based transition care services. However, this was a retrospective study using medical case files and there were limited outcome measures available for retrieval. The audit also identified that there was a gap in measuring other health outcomes that would be helpful to evaluate during rehabilitation such as mental and emotional well-being. Other research has also suggested that more comprehensive assessments should be completed in TCP to understand changes in older adults' health and wellbeing (3). Further prospective studies that evaluate a broad range of functional outcomes such as in a comprehensive geriatric assessment are required to assist in evaluating the effectiveness of TCP. A recent study suggested that older adults who completed home based TCP demonstrated better health outcomes compared to those who completed facility-based TCP (3). As only one setting was examined, further studies are required to investigate differences between settings and countries to determine how to make best use of scarce resources for older adults who require transition care after hospital discharge.

## Conclusions

An audit identified that older adults admitted to a facility-based TCP were significantly more likely to be discharged home if they were independent with ADL at admission, had an orthopedic diagnosis, had good levels of cognition, and had a pre-planned team goal to be discharged home. Those cases discharged home, made significantly more improvement in performance of ADL than those discharged elsewhere. Older adults with cognitive impairment had lower levels of functional ability on TC admission, however there were no significant differences in the magnitude of improvement in functional ability between these older adults and those without cognitive impairment. Older adults with cognitive impairment were significantly more likely to be discharged to RAC.

Future research that compares facility-based TCP with other forms of TCP would assist in determining the most effective means of providing older adults with transition care after hospitalization.

## Data Availability Statement

The datasets presented in this article are not readily available because due to the organization's privacy policy, a deidentified dataset could only be provided if suitable ethics approvals are obtained. Requests to access the datasets should be directed to Professor Anne-Marie Hill; Anne-Marie.Hill@curtin.edu.au.

## Ethics Statement

The studies involving human participants were reviewed and approved by Curtin University Human Research Ethics Committee and Amana Living Inc's clinical governance committee. Written informed consent for participation was not required for this study in accordance with the national legislation and the institutional requirements.

## Author Contributions

J-AH led the drafting of the manuscript with support from JF-C, CN, and A-MH. J-AH, JF-C, and A-MH led the research design with support from NW and KP, and assisted with monitoring the research. J-AH and A-MH led the research with support from JF-C and NW. J-AH collected data at the designated site and KP led management of the research at the facility. CN and AJ contributed to statistical analysis. J-AH undertook this research project as part of her doctoral studies under the guidance of JF-C, NW, and A-MH. All authors provided critical evaluation and approval of the final submitted manuscript.

## Conflict of Interest

KP is employed by Amana Living Inc. organization. The remaining authors declare that the research was conducted in the absence of any commercial or financial relationships that could be construed as a potential conflict of interest.

## Abbreviations

ADL: activities of daily living
AOR: adjusted odds ratio
A&F: audit and feedback
CI: confidence intervals
IRSAD: Index of Relative Socio-Economic Advantage and Disadvantage
IADL: Instrumental Activities of Daily Living
IQR: interquartile ranges
LOS: length of stay
MCID: Minimal Clinically Important Difference
MBI: Modified Barthel Index
OR: odds ratio
RAC: residential aged care
TCP: transition care programs
TC: transition care.
