# Supplementary material for: Identifying the Association Between Older Adults' Characteristics and Their Health-Related Outcomes in a Transition Care Setting: A Retrospective Audit
Source: Front Public Health. 2021 Jun 28;9:688640. doi: 10.3389/fpubh.2021.688640 (PMC8294153; doi:10.3389/fpubh.2021.688640)
Supplement: Supplementary file 1 [file Data_Sheet_1.docx]

**SUPPLEMENTARY FILE**

|  | **Title** | **Page number** |
| --- | --- | --- |
| eTable 1 | Case characteristics on admission to Transition Care facility. | 2 |
| eTable 2 | Multivariable analysis for Outcome = Discharge home. | 4 |
| eFigure 1 | Adjusted predictions of MBI for cases’ age group and cognitive group. | 6 |

**eTable 1.** Case characteristics on admission to Transition Care facility.

| Characteristic | Cases, *n(%),* n=169 |
| --- | --- |
| Age, mean (SD) | 84.2 (8.3) |
| Age 60-79 years | 50 (29.6) |
| Age ≥80 years | 119 (70.4) |
| Gender, female | 103 (60.9) |
| Socioeconomic status, IRSAD^†^ |  |
| I | 15 (8.9) |
| II | 49 (29.0) |
| III | 105 (62.1) |
| Hospital length of stay prior to TC admission (days), median (IQR) | 33.0 (21.0-49.0) |
| TC length of stay (days), median (IQR) | 38.0 (21.0-70.0) |
| Pre-hospital admission living situation |  |
| Lived alone | 97 (50.5) |
| With family | 72 (49.5) |
| Uses walking aid |  |
| Frame or walker | 122 (72.2) |
| Walking stick or quad cane or elbow crutch | 10 (5.9) |
| Non-ambulant | 25 (14.8) |
| Primary medical diagnosis |  |
| Neurological (includes dementia) | 25 (14.8) |
| Cardiorespiratory | 30 (17.8) |
| Orthopaedic (includes hip fracture) | 56 (33.1) |
| General medicine and surgery | 28 (16.6) |
| Geriatric related^‡^ (such as poor balance, or frailty) | 30 (17.8) |
| Cognitive score at admission,^§^ mean (SD) | 21.4 (5.5) |
| Scored≤23/30 at admission^¶^ | 73 (47.7) |
| Mental health diagnosis^††^ | 34 (20.1) |
| Number of medications on admission |  |
| Up to 6 medications | 30 (18.1) |
| 7 to 12 medications | 81 (48.8) |
| 13 or more medications | 55 (33.1) |
| Falls history prior to TC admission |  |
| No fall | 28 (17.0) |
| 1 fall | 56 (33.9) |
| Multiple (two or more) falls | 81 (49.1) |
| Presence of depressive symptoms^‡‡^ | 61 (62.9) |
| Malnourishment^§§^ | 98 (70.5) |
| Notes: SD, Standard Deviation; IQR, Interquartile Range; TCP, Transition Care Programs; TC, Transition Care; All data are reported as n (%) unless otherwise stated. Where data not = 100% data are missing. | |
| ^†^IRSAD = The Index of Relative Socio-Economic Advantage and Disadvantage 2016, where I = most disadvantaged socioeconomic area (decile 1 to 4) and III = most advantaged socio-economic area (decile 9 and 10) | |
| ^‡^Includes poor balance, malnutrition, frailty, polypharmacy, incontinence, delirium and fall risk (17)  ^§^Measured using Mini Mental State Examination (MMSE), range 0-30, higher point indicates better cognitive function, score of 23 or below indicates presence of cognitive impairment (18, 19) | |
| ^¶^Classified as having cognitive impairment | |
| ^††^Includes secondary diagnosis of depression, anxiety, suicidal ideation, post-traumatic stress disorder, low mood, adjustment disorder and paranoia | |
| ^‡‡^Measured using Geriatric Depression Scale, range 0-15 points, higher points indicate presence of depression, score of 6 or more indicates presence of depression (20) | |
| ^§§^Measured using Mini Nutritional Assessment Short-Form (MNA-SF), score range 0-14, high points indicate normal nutritional status, score of 11 or below indicates at risk of malnourishment or malnourishment (21) | |

**eTable 2.** Multivariable analysis for Outcome = Discharge home.

| Independent Variable | Univariable - Association with discharge home | | | Multivariable - Association with discharge home | | |
| --- | --- | --- | --- | --- | --- | --- |
|  | OR | 95% CI | P value^†^ | Adjusted OR | 95% CI | P value |
| Age | 0.96 | 0.93 – 1.00 | 0.061 |  |  |  |
| Gender | 0.80 | 0.42 – 1.56 | 0.518 |  |  |  |
| Socioeconomic status, IRSAD^‡^ | 0.63 | 0.30-1.31 | 0.214 |  |  |  |
| Assistance with |  |  |  |  |  |  |
| ADL | 0.47 | 0.23 – 0.96 | 0.038^*^ | 0.41 | 0.16-1.00 | 0.049 |
| IADL | 1.00 | 0.38 – 2.64 | 0.988 |  |  |  |
| Used of walking frame | 0.97 | 0.53 – 1.79 | 0.934 |  |  |  |
| Lived alone | 0.74 | 0.39 – 1.44 | 0.380 |  |  |  |
| Hospital LOS | 0.99 | 0.98 – 1.00 | 0.169 |  |  |  |
| Performance of ADL | 1.01 | 0.998 – 1.03 | 0.082 |  |  |  |
| 7+ medications | 1.62 | 0.65 – 4.07 | 0.300 |  |  |  |
| Primary diagnoses |  |  |  |  |  |  |
| Neurological | 0.63 | 0.24 – 1.68 | 0.359 |  |  |  |
| Cardiorespiratory | 0.19 | 0.06 – 0.66 | 0.009^*^ |  |  |  |
| Orthopedic | 3.35 | 1.69 – 6.62 | 0.001^*^ | 3.63 | 1.51-8.68 | 0.004 |
| General medicine/surgical | 1.22 | 0.52 – 2.87 | 0.641 |  |  |  |
| Geriatric | 0.60 | 0.24 – 1.49 | 0.268 |  |  |  |
| Mental health diagnosis^§^ | 0.72 | 0.31 – 1.67 | 0.444 |  |  |  |
| History of falls | 4.79 | 1.37– 16.67 | 0.014^*^ |  |  |  |
| Presence of cognitive impairment^¶^ | 0.43 | 0.22 – 0.86 | 0.017^*^ | 0.41 | 0.18-0.93 | 0.033 |
| Incontinence | 0.66 | 0.33 – 1.29 | 0.220 |  |  |  |
| Presence of depressive symptoms^††^ | 0.90 | 0.37 – 2.18 | 0.824 |  |  |  |
| Malnourished^‡‡^ | 1.45 | 0.68 – 3.10 | 0.338 |  |  |  |
| Discharged to pre-planned discharge destination | 5.51 | 2.68 – 11.32 | <0.001^*^ | 24.98 | 5.47-114.15 | <0.001 |
| Notes: ADL, activities of daily living; CI, confidence interval; IADL, instrumental activities of daily living; LOS, length of stay; OR, odds ratio | | | | | | |
| ^†^Independent variables that demonstrated statistical significance were entered into the multivariate model. All variables that remained statistically significant in the final model were retained. | | | | | | |
| ^‡^IRSAD = The Index of Relative Socio-Economic Advantage and Disadvantage 2016, where I = most disadvantaged socioeconomic area and III = most advantaged socio-economic area | | | | | | |
| ^§^Co-morbidities, includes depression, anxiety, suicidal ideation, post-traumatic stress disorder, low mood, adjustment disorder and paranoia | | | | | | |
| ^¶^Measured using Mini Mental State Examination (MMSE), scored≤23/30 at admission (18, 19) | | | | | | |
| ^††^Measured using Geriatric Depression Scale, scored≥6 points indicating presence of depression (20) | | | | | | |
| ^‡‡^Measured using Mini Nutritional Assessment Short-Form (MNA-SF), scored ≤11 points indicating malnourishment (21) | | | | | | |

**FIGURE LEGENDS**

**eFigure 1.** Adjusted predictions of MBI for cases’ age group and cognitive group.
