# Supplementary figures and images for: Identifying the Association Between Older Adults' Characteristics and Their Health-Related Outcomes in a Transition Care Setting: A Retrospective Audit
Source: Front Public Health. 2021 Jun 28;9:688640. doi: 10.3389/fpubh.2021.688640 (PMC8294153; doi:10.3389/fpubh.2021.688640)

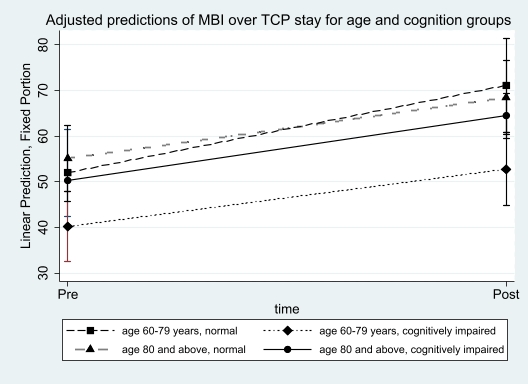

Supplement: Supplementary file 2 [file Image_1.JPEG]
